# Nutrient Sufficiency Ranges for Corn at the Early Growth Stage: Implications for Nutrient Management

**DOI:** 10.3390/plants12040713

**Published:** 2023-02-06

**Authors:** Solomon Amissah, Godfred Ankomah, Benjamin K. Agyei, Robert D. Lee, Glendon H. Harris, Miguel Cabrera, Dorcas H. Franklin, Juan C. Diaz-Perez, Mussie Y. Habteselassie, Henry Y. Sintim

**Affiliations:** 1 Department of Crop & Soil Sciences, University of Georgia, Tifton, GA 31793, USA; 2 Department of Plant, Soil, and Microbial Sciences, Michigan State University, East Lansing, MI 48824, USA; 3 Department of Crop & Soil Sciences, University of Georgia, Athens, GA 30602, USA; 4 Department of Horticulture, University of Georgia, Tifton, GA 31793, USA; 5 Department of Crop & Soil Sciences, University of Georgia, Griffin, GA 30223, USA

**Keywords:** plant nutrition, tissue analyses, sufficiency range system, nutrient imbalance, corn biomass

## Abstract

Growers rely on nutrient sufficiency ranges (NSRs) after plant tissue analysis to inform timely nutrient management decisions. The NSRs are typically established from survey studies across multiple locations, which could be confounded by several abiotic and biotic factors. We conducted field studies in 2020, 2021, and 2022 to validate the lower thresholds of the NSRs for corn (*Zea mays*) at the early growth stage as reported in the Southern Cooperative Series Bulletin #394. We induced various corn nutritional levels by making different nutrient application rates. If the NSRs are valid, samples within the same replication that satisfy the NSRs of all nutrients should have similar biomass accumulation. The results showed that the NSRs were not valid under the conditions tested. In total, 47.6% of the samples satisfied all the lower thresholds of the NSRs, and 25.4% of those samples had relative biomass <50%, with relative biomass even as low as 24.2% observed. Moreover, 9.6% of the total samples had P and Cu levels that failed to meet the lower threshold but still had relative biomass ≥75%. The findings highlight the sensitivity of corn to nutrient imbalance and the need to optimize nutrient diagnostic methods at the early growth stage.

## 1. Introduction

Nutrient elements are indispensable for plant growth because they support critical metabolic functions in plants [1,2,3]. Without essential nutrients, plants cannot complete their life cycle [2,4,5]. Moreover, the functions of essential nutrients are specific, indicating that the surplus of one nutrient element cannot substitute the deficiency of another nutrient element, as depicted by the “Law of the Minimum” and the “Law of the Optimum” [5,6,7,8]. Maintaining adequate and balanced plant nutrition is crucial for crops to attain their genetic growth potential [9,10,11].

As a high-input crop, corn (*Zea mays*) requires substantial nutrients and it is also susceptible to nutrient imbalance [9,12,13]. Imbalanced nutrient supply affect nutrient uptake, utilization, and overall corn productivity [9,14]. Plant tissue analysis is a valuable method to monitor the nutritional health of crops. It complements soil tests to provide insight into the nutrient levels of plants at the time of sampling for timely nutrient management decisions [10,15]. An appropriate and convenient interpretation of plant tissue analysis results is needed for tissue analysis to be helpful as a management tool. Several methods are available to interpret plant tissue analysis results, which can be grouped into two major categories: (a) independent nutrient indices and (b) dependent nutrient indices [10].

A classic example of the independent nutrient indices is the sufficiency range system (SRS), an extension of the critical level approach (CLA). The CLA provides a single value for every relevant nutrient, set at 90% to 95% of the maximum growth or yield of a nutrient response model [16,17]. The SRS offers a significant advantage over the CLA by setting lower and upper threshold values for relevant nutrients, which indicate the optimum nutrient concentration levels to obtain maximum growth or yield. As nutrient concentration in plant tissues changes over the growing season, different threshold values are typically set in SRSs for the various growth stages. A classic example of the dependent nutrient indices is the Diagnostic and Recommendation Integrated System (DRIS), which calculates the ratio of all possible pairs of relevant nutrients and compares the ratios to that of high-yielding crops [9,18,19]. Standard scores are computed for each nutrient and averaged to obtain one index per nutrient. The DRIS prioritizes the relationship among nutrients, rather than absolute concentrations, and ranks the nutrients in their order of limitedness. Baldock and Schulte [10] proposed the Plant Analysis with Standardized Scores system, which integrated both the SRS and DRIS.

The SRS is one of the most common interpretative methods used by farmers because of its convenience and ease of adoption [16,20]. The lower and upper threshold values for every nutrient in SRSs mainly were based on subjective interpretation of plant analyses and survey studies across multiple locations [20,21], which can be confounded by several abiotic and biotic factors that impact crop growth and productivity [22,23]. For instance, the mineralization of nutrients and subsequent crop uptake can be affected by rainfall, temperature, type of crop residues, and soil properties, such as the pH, aeration, and the structure and composition of microbial communities [24,25,26,27,28]. Thus, several reports have surmised that established threshold values in SRSs are not all-inclusive and absolute [9,10,29], but that needs to be validated.

The Southern Cooperative Series Bulletin (SCSB) #394 is a popular and valuable output of a regional project that provides nutrient sufficiency ranges (NSRs) for plant analyses in the southern region of the United States [30]. The NSRs, like any other, have not been validated in controlled experiments. Therefore, the objective of this study was to validate the lower threshold of the NSRs for corn at the early growth stages. The NSRs for corn at the early growth stage as reported in the SCSB #394 are 30–40 g kg^−1^, 3–5 g kg^−1^, 20–30 g kg^−1^, 2.5–8 g kg^−1^, 1.5–6 g kg^−1^, 1.5–4 g kg^−1^, 30–250 mg kg^−1^, 20–150 mg kg^−1^, 20–70 mg kg^−1^, 5–25 mg kg^−1^, 5–25 mg kg^−1^, and 0.1–2 mg kg^−1^, respectively, for N, P, K, Ca, Mg, S, Fe, Mn, Zn, Cu, B, and Mo [30]. The early growth stage for corn in SCSB #394 represents plants that are >10 cm in height to tasseling.

A controlled experiment is needed to validate the threshold values, and all relevant nutrients must be validated simultaneously, which is very complicated to accomplish. To date, there is no management system to have nutrients accumulate precisely at specific levels. As plant nutrient uptake is regulated by several factors [2,31], there is no guarantee that plant tissues sampled at a particular stage will have all the nutrients within the sufficiency ranges, regardless of the nutrient management regime. Based on the explicit meaning of the NSR, we posit that plant samples with all nutrients within the sufficiency ranges should accumulate similar biomass under a controlled field experiment, where nutrient levels are the only variables. We note that it would be a wrong inference to use yield to validate the sufficiency ranges at the early growth stage because yield can be confounded by several variables post-sampling at the early growth stage. Moreover, it would be practically impossible to maintain those nutrient levels until harvest.

Field experiments were set up in 2020, 2021, and 2022 to assess the lower threshold of the NSRs for corn at the early growth stage as reported in the SCSB #394. Corn plants were maintained at various nutritional levels by imposing different fertilizer application regimes (Table 1) which constituted the experimental treatments. Plant tissue analyses were performed, along with measuring plant biomass. If the NSRs reported in the SCSB #394 are valid, we expected samples with all nutrient levels within the NSRs to have ~100% relative biomass.

## 2. Results and Discussion

### 2.1. Weather and Initial Soil Nutrients

The average minimum, mean, and maximum temperatures, as well as the total growing degree days (GDD), solar radiation, and rainfall received from planting to tissue sampling each year are reported in Table 2. The weather was relatively warmer in 2021 compared to 2020 and 2022, as shown by the greater minimum, mean, and maximum air temperature, and the total GDD and solar radiation. Whereas temperature and GDD were greater in 2020 than in 2022, solar radiation was greater in 2022. Total rainfall during the growing period in the three years varied considerably, with 2021 receiving the highest rainfall and 2022 the least rainfall. 

The initial nutrient concentration of the soil varied with depth over the three years (Table 3). In general, there was nutrient stratification with the primary and secondary nutrients being concentrated in the top 0–15 cm depth than in the 15–30 cm depth, except in 2022, where the reverse was observed in P. Stratification of micronutrients was not very conspicuous, except in 2021 where more than 2-fold of Cu concentration was observed in the 15–30 cm depth as compared to that observed at the top 0–15 cm depth. For the primary nutrients, N and P were greatest in 2020, whereas 2022 had the highest K level at the top 0–15 cm depth, and 2021 had the greatest K level at the 15–30 cm depth. Mg was greatest at both depths in 2022. Ca was substantially low at both depths in 2021 and the 15–30 cm depth in 2020. S was greatest in 2021 and 2022 at 0–15 cm and 15–30 cm depths, respectively. For the micronutrients, B was the least in 2021, and Zn and Mn were the least in 2022. Fe and Cu were substantially high in 2022 and 2021, respectively.

### 2.2. Validation of Reference Sufficiency Thresholds

**Figure 1:**
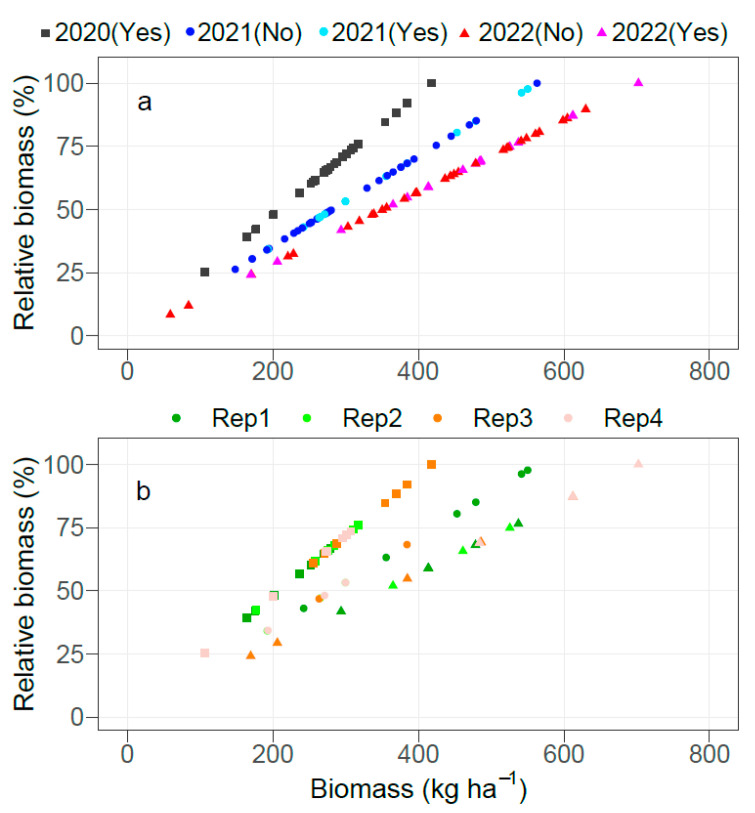
Scatter plot of relative biomass vs. biomass of corn sampled at the V6-V7 growth stage in 2020, 2021, and 2022. In (**a**), “Yes” refers to samples with all nutrient concentrations satisfying the lower thresholds of the reference sufficiency ranges for corn at the early growth stage in the Southern Cooperative Series Bulletin #394, whereas “No” refers to samples with at least one nutrient below the lower threshold; (**b**) shows only samples denoted as ‘Yes’ in (**a**), and the shapes in (**b**) denote the different years as noted for (**a**).

A scatter plot of relative biomass vs. biomass is provided in Figure 1. In Figure 1a, samples with all nutrient concentrations satisfying the lower threshold of the reference sufficiency ranges for corn at the early growth stage in the SCSB are denoted as ‘Yes’, and ‘No’ for samples with at least one nutrient below the lower threshold. Figure 1b shows only samples denoted as ‘Yes’ in Figure 1a and differentiates the replications. The biomass ranged from 106 kg ha^−1^ to 418 kg ha^−1^ in 2020, from 148 kg ha^−1^ to 563 kg ha^−1^ in 2021, and from 59.2 kg ha^−1^ to 702 kg ha^−1^ in 2022. In general, there was a good distribution of samples across the entire biomass range in all three years. All samples satisfied the lower threshold of the NSRs in 2020, and 29% and 35% of samples satisfied the lower threshold in 2021 and 2022, respectively.

Within every replication, we expected samples that satisfied all the lower thresholds of the NSRs to have similar relative biomass unless (a) the lower thresholds of the NSRs were not valid or (b) there was a toxic level of a nutrient that impaired growth. The sufficiency range indicates the optimum level to achieve maximum growth, and additional nutrient accumulation could result in toxicity levels where plant growth is impacted [32,33,34]. Samples that satisfied the lower threshold of the NSRs had wide variations in relative biomass. In 2020, the minimum vs. maximum relative biomass within replications 1, 2, 3, and 4 were 39.2% vs. 66.8%, 42.3% vs. 75.9%, 61.0% vs. 100%, and 25.4% vs. 73.5%, respectively. In 2021, the minimum vs. maximum relative biomass within replications 1, 2, 3, and 4 were 43.1% vs. 97.7%, 34.2% vs. 53.2%, 46.8% vs. 68.3%, and 34.3% vs. 53.3%, respectively. In 2022, the minimum vs. maximum relative biomass within replications 1, 2, 3, and 4 were 41.8% vs. 76.5%, 52.0% vs. 74.9%, 24.2% vs. 87.2%, and 68.9% vs. 100%, respectively.

To ascertain potential toxicity effects, we compared the nutrient concentrations of samples that satisfied all the lower thresholds of the NSRs and had the minimum relative biomass in every replication with those with greater relative biomass. Of the 12 total samples (one per replication over three years), three had at least one nutrient that was the greatest within the replication, suggesting potential toxicity effects: treatment T7 in replications 2 (N, B, and Zn) and 3 (B) in 2020, and treatment T4 in replication 3 (Zn, Mn, and Cu) in 2021. The remaining nine samples, however, suggest that the lower threshold may not be valid. Moreover, 12 samples had P or Cu levels that failed to meet the lower threshold but still had relative biomass ≥75%. In 2021, treatment T2 of replication 1 had a Cu level of 4.0 mg kg^−1^, below the lower threshold of 5.0 mg kg^−1^, but it had relative biomass of 100%. In 2022, treatment T4 of replication 4 had a P level of 2.6 g kg^−1^, below the lower threshold of 3.0 g kg^−1^, but it had relative biomass of 86.1%. Additionally, the P (2.9 g kg^−1^) and Cu (4.0 mg kg^−1^) levels of treatment T4 of replication 2 were below the lower threshold, but it still had relative biomass of 85.2%. Treatments T5 and T11 had Cu levels as low as 2.0 mg kg^−1^ but had relative biomass of 80.6% and 78.1%, respectively. The findings of this study suggest that either the lower thresholds of Cu and P are smaller than recommended, or they were not the most limited. They further highlight the inability of the SRS to account for nutrient interactions and the magnitude of the limitation of each nutrient [9,10,18].

### 2.3. Nutrient Dynamics

**Figure 2:**
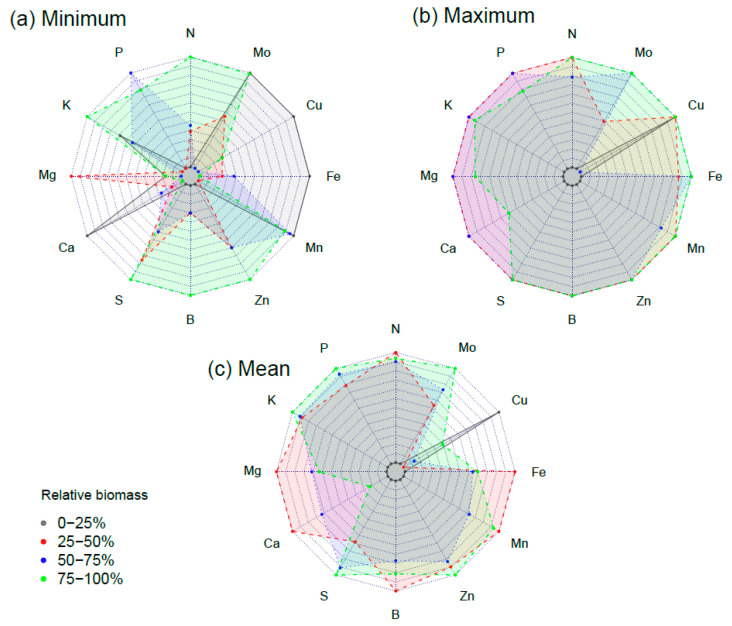
Radar plots of the minimum, maximum, and mean relative nutrient concentrations of corn samples with 0–25%, 25–50%, 50–75%, and 75–100% relative biomass.

Radar plots of the minimum, maximum, and mean relative nutrient concentrations of samples with 0–25%, 25–50%, 50–75%, and 75–100% relative biomass are shown in Figure 2. Samples with 75–100% relative biomass had the greatest minimum N, K, S, B, and Zn levels (Figure 2a), suggesting that higher basal levels of those nutrients are needed to sustain good plant growth [11,35]. The samples with 75–100% relative biomass, however, had low minimum Mg, Ca, Fe, and Cu levels (Figure 2a). In contrast, samples with 0–25% relative biomass had distinctively greatest minimum Ca, Fe, and Cu levels, whereas those with 25–50% relative biomass had distinctively greater minimum Mg levels. The maximum Mg, Ca, Fe, and Cu levels were, however, also high in samples with 50–75% and 75–100% relative biomass (Figure 2b), suggesting nutrient deficiency or imbalance rather than toxicity of those nutrients, could explain the poorer growth in samples with 0–25% and 25–50% relative biomass. The mean nutrient levels were high for samples with 75–100% relative biomass, except for Ca and Cu (Figure 2c). Samples with 0–25% relative biomass had the lowest mean nutrient levels, except for Cu where it had the greatest concentration.

Table 4 shows the summary statistics of the nutrients of samples with 95–100% relative biomass. Except for P and Cu, the minimum levels of the nutrients amongst samples with 95–100% relative biomass (Table 4) were greater when compared to the lower threshold of the NSRs for corn at the early growth stage in the SCSB. The maximum levels of N, P, K, S, B, and Mo of samples with 95–100% relative biomass were greater than the upper threshold of the NSRs for corn at the early growth stage in the SCSB. However, the samples with 95–100% relative biomass had lower Ca, Zn, Mn, Fe, and Cu than the upper threshold.

Pioneer conducted on-farm corn tissue sampling research in the United States and Canada in 2018, 2019, and 2020 [20]. The study used 550 on-farm trials with an average yield of 16.9 to 29.8 Mg ha^−1^ to establish NSRs for corn at the V6-V8, VT-R1, and R3-R5 growth stages. The V6-V8 NSRs derived for N, P K, Mg, Ca, S, B, Zn, Mn, Fe, and Cu were 38.0–41.0 g kg^−1^, 3.3–3.8 g kg^−1^, 29.7–33.8 g kg^−1^, 2.0–2.6 g kg^−1^, 4.7–5.7 g kg^−1^, 2.4–2.8 g kg^−1^, 10.2–14.3 mg kg^−1^, 31.0–39.7 mg kg^−1^, 66.8–94.9 mg kg^−1^, 168–207 mg kg^−1^, and 11.0–15.5 mg kg^−1^, respectively [20]. The sufficiency ranges reported by Blessitt [20] are within the minimum and maximum nutrient levels of samples with 95–100% relative biomass in this study, except for B, Fe, and Cu. The lower and upper thresholds of B reported by Blessitt [20] were lower than the minimum level observed in samples with 95–100% relative biomass in this study, whereas the upper thresholds of Fe and Cu reported by Blessitt [20] were greater when compared to the maximum levels observed in samples with 95–100% relative biomass.

**Figure 3:**
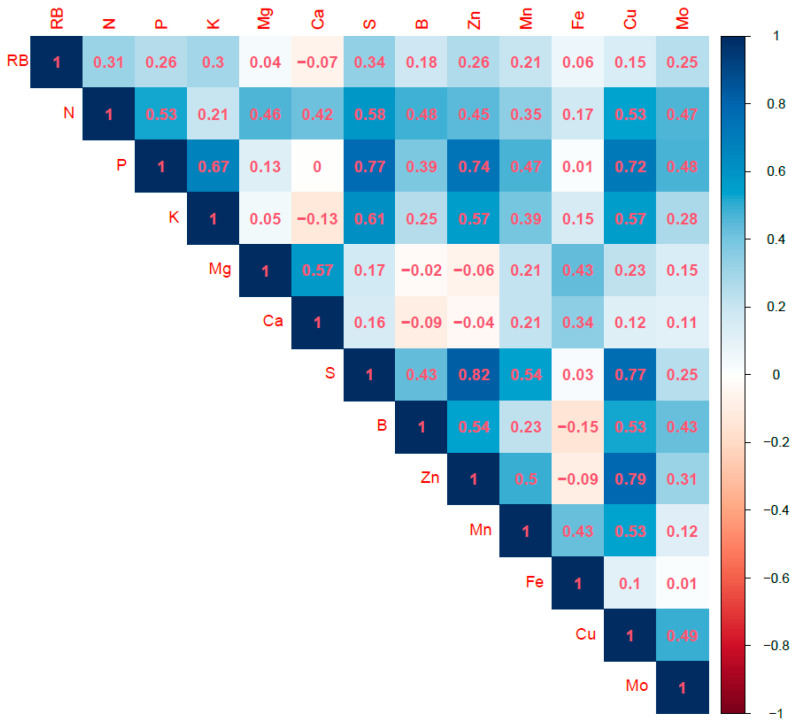
Pearson correlation coefficients of relative biomass (RB) and nutrient concentrations.

The relative biomass had significant correlations with N, P, K, S, B, Zn, Mn, and Mo; however, the correlations were generally weak (Table 5 and Figure 3), indicating that an increased concentration of plant nutrients does not necessarily lead to improved plant growth [9,32]. The correlation of relative biomass between only N, K, and S yielded Pearson correlation coefficients ≥0.3. No significant negative correlation was observed between relative biomass and nutrients. Kovács and Vyn [35] reported significant positive correlations between corn ear-leaf nutrient levels and biomass sampled at the mid-silking stage, except for B, which yielded a significant negative correlation with biomass. However, the correlation was weak with a Pearson correlation coefficient of −0.128 [35].

More significant correlations of the plant nutrients were observed, consistent with the findings of other studies [11,35]. In general, N and Mn had the maximum number of significant correlations with other nutrients (Table 5). N and Mn had significant correlations with all nutrients except N vs. Fe and Mn vs. Mo correlations that were not significant. Cu had significant correlations with nine other nutrients, and P, K, S, B, and Zn had significant correlations with eight other nutrients. The strongest positive correlation was between S and Zn, with a Pearson correlation coefficient of 0.82. There was no significant correlation between nutrients with a negative Pearson correlation coefficient, consistent with the observations of Bojtor et al. [11].

### 2.4. Treatment Effects on Biomass and Nutrient Concentration

**Figure 4:**
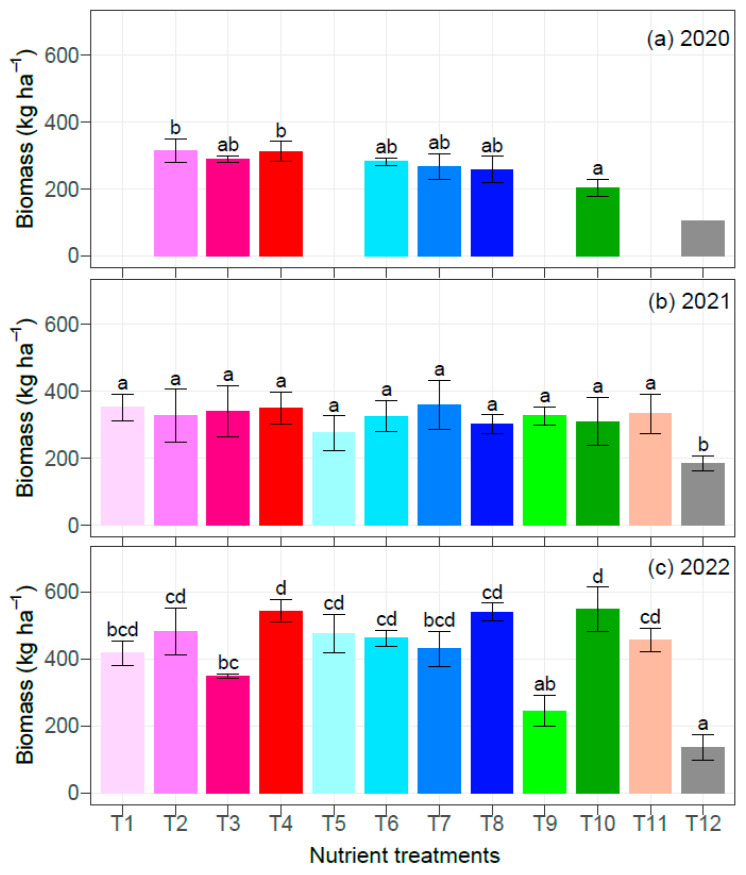
Nutrient treatment effects on biomass of corn from 2020 to 2022. Within a study year, means not sharing any letter are significantly different using the least squares means and adjusted Tukey multiple comparisons (*p* < 0.05). Treatment T12 in 2020 had one replication, and so it was excluded from the statistical analyses. Error bars indicate the standard deviation of the mean (n = 4).

The nutrient rates had significant effects on the biomass (*p* = 0.027 in 2020; *p* = 0.01 in 2021; and *p* < 0.001 in 2022), and, as expected, treatment T12 (no nutrient application) consistently had the least biomass over the three years (Figure 4). Treatments T1, T5, T9, and T11 were not assessed in 2020, and there was only one replication for treatment T12, which was excluded from the statistical analyses. In general, increasing rates of N, P_2_O_5_, and K_2_O (T1 to T4 vs. T5 to T8) did not significantly increase biomass. In 2022, treatment T3 biomass was significantly lower than that of treatment T4. Treatment T3 did not receive any secondary nutrients, but it had similar rates of primary and micronutrients as treatment T4, highlighting the importance of secondary nutrients in corn growth [36,37,38]. As already noted, treatment T10 received greater rates of all nutrients than treatments T4 and T8. However, it had similar biomass as those of treatments T4 and T8, except in 2020 when it had significantly lower biomass than treatment T4, and the biomass of treatment T8 was intermediate. The results in 2020 could be attributable to toxicity effects; the nutrient application rates of treatment T10 in 2020 were generally greater than those in 2021 and 2022 (Table 1). Nonetheless, in 2020, there was no single nutrient in treatment T10 where the concentration was overly high compared to any sample (Table 6). The level of B in 2020 seemed to be high (66.3 mg kg^−1^), but a similar value (66.2 mg kg^−1^) was observed in 2022.

The different nutrient rates applied also significantly affected plant tissue nutrient levels, but the trend was not consistent (Table 6). The high nutrient rates applied in treatment T10 in 2020 led to significantly greater N levels than those in treatments T2 and T7; however, no significant differences in N levels were observed between treatments in 2021, except for treatment T12. In 2022, treatments that received all forms of nutrients (treatments T4, T8, and T10) tended to have increased N levels. Differences in P levels were marginal in 2021 but significant in 2020 and 2022. In addition, the differences in K levels were marginal in 2020 and 2021 but significant in 2022. The lack of response in P levels in 2021 and K levels in 2020 and 2021 was unexpected as the initial soil levels were not overly high in P and K. For instance, the P level in 2020 was higher than in 2021, and also the K level in 2022 was greater than in 2020 and 2021. Ca levels were not affected by the different nutrient rates in all three years, and the Mg levels were not affected by the different nutrient rates in 2021 and 2022. The Mg level was significantly reduced in treatment T10 compared to treatment T2 in 2020. The effect of nutrient application rates on S was observed in all three years, with the impact being more evident in 2022. The S level was highest in treatment T8 and least in treatment T12. Significant differences in the micronutrient levels as a result of the nutrient application rates were observed, except for Mn and Fe in 2020 and 2021. In general, the micronutrients accumulated more in treatments that received micronutrient application.

The marginal responses in corn biomass and nutrient levels to increased nutrient application rates could be attributed to low nutrient demand for corn at the early growth stage. It is estimated that <15% of nutrients are typically taken up by the V6 to V7 growth stage compared to the total nutrients taken up at physiological maturity [39,40,41]. These findings suggest there is potential to synchronize nutrient applications with plant demand, maximizing productivity and nutrient use efficiency in corn [39,42,43].

## 3. Practical Implications

**Figure 5:**
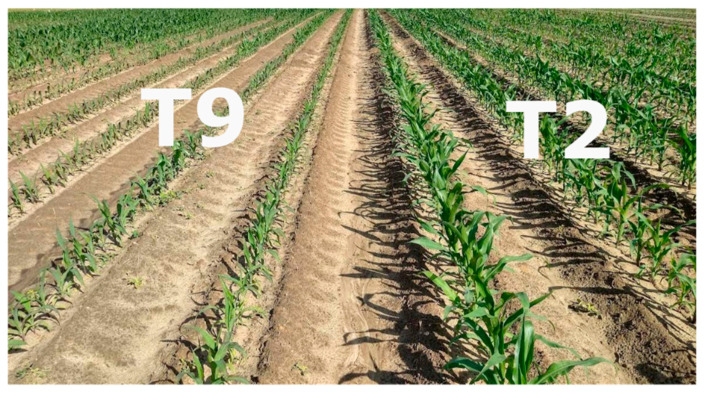
Photo of the experimental field in 2022 showing corn plants in treatments T2 (healthy crops) and T9 (stunted crops). Both the stunted and healthy crops had all nutrient levels that satisfied the lower threshold of the nutrient sufficiency ranges for corn at the early growth stage reported in the Southern Cooperative Series Bulletin #394.

Figure 5 shows corn plants in treatments T2 and T9 exhibiting healthy and stunted growth, respectively. The healthy and stunted crops had all the nutrient levels satisfying the lower threshold of the SCSB’s NSRs for corn at the early growth stage. Without the healthy plants, a grower would think there was nothing wrong with the stunted crops given that the foliage was still green. More importantly, the NSRs of the SCSB would have also not been able to detect issues with the crop, given that the levels of all the nutrients satisfied the NSRs. Further studies to ascertain whether corn can fully recover from early season nutrient stress would help inform nutrient management decisions.

## 4. Materials and Methods

### 4.1. Experimental Site

The study was conducted at the University of Georgia Coastal Plains Experiment Station in Tifton, GA (31°31′06.5″ N, 83°32′59.6″ W). Tifton has a humid subtropical climate characterized by relatively high temperatures and evenly distributed precipitation. The annual average daily minimum, mean, and maximum air temperatures are 12.7, 19.0, and 25.2 °C, respectively, and the average annual precipitation is 1208 mm with an average of 109 rainy days [44]. The experimental field had a Dothan sandy loam soil classified as fine-loamy, kaolinitic, thermic Plinthic Kandiudults. The soil had sand, silt, and clay content of 91.9%, 4.5%, and 3.6% at the top 0–15 cm depth, respectively, and 87.9%, 4.5%, and 7.6% at the 15–30 cm depth, respectively. Further, the soil pH and organic matter were 6.4 and 4.8 g kg^−1^ at the top 0–15 cm depth, respectively, and 6.5 and 4.4 g kg^−1^ at the 15–30 cm depth, respectively.

### 4.2. Field Experiment

Field experiments in 2020, 2021, and 2022 evaluated early-season corn growth impacted by various nutritional levels. The nutritional levels were induced by different nutrient application rates, which constituted the treatments (Table 1). Twelve treatments were assessed in 2021 and 2022, and eight treatments in 2020. Every year, Treatments T1 to T4 had similar N, P, and K (referred hereafter as primary nutrients) application rates, except that T2 received additional Mg, Ca, and S (referred hereafter as secondary nutrients), and T3 received additional B, Zn, Mn, Fe, Cu, and Mo (referred hereafter as micronutrients). Treatment T4 received both secondary and micronutrients. Treatments T5 to T8 received similar N, P, and K rates, but the rates were greater than those in treatments T1 to T4. Additionally, treatment T1 corresponded to T5, T2 to T6, T3 to T7, and T4 to T8 regarding secondary and micronutrient application rates. Treatment T10 received primary, secondary, and micronutrients at greater rates than T8, whereas treatment T12 was a control, receiving no nutrient application. Treatments T9 and T11 were not assessed in 2020 but varied in 2021 and 2022. Treatment T9 received all primary, secondary, and micronutrients in 2021 and only N and P in 2022. Treatment T11 received N, P, K, and S in 2021 and N, P, K, S, and Zn in 2022. Different nutrient rates were used yearly based on initial nutrient levels, previous crops, and observations of the results from preceding years. Fertilizers as the primary sources of the different nutrients are listed in Table 7.

The treatments were laid out in a randomized complete block design with four replications, except for treatment T12 in 2020, which had only one replication embedded in the fourth replication. Each experimental plot was 9.1 m long and 5.5 m wide. Different research fields at the experimental site were used yearly; thus, treatment effects over the three years were not additive. The previous crop for the years 2020 and 2022 was peanut (*Arachis hypogaea*) and for the year 2021 it was cotton (*Gossypium hirsutum*). The field was prepared by harrowing, running a field cultivator, and making 20 cm deep furrows that were 91.4 cm apart. Pioneer hybrid P1870R was used every year and it was planted at 88,958 seeds ha^−1^ on 7 April, 12 April, and 25 March in 2020, 2021, and 2022, respectively. Glyphosate [N-(phosphonomethyl) glycine], Atrazine (2-chloro-4-ethyl amino-6-isopropyl amino-s -triazine), and Prowl [N-(1-ethyl propyl)-3,4-dimethyl-2,6-dinitrobenzenamine] were applied at manufacturer recommended rates to control weeds in the corn plots between the V3 and V4 growth stage. The plots were provided with supplemental water through overhead irrigation whenever the soil moisture reached 50% of the water-holding capacity of the soil.

### 4.3. Data Collection

Soil samples were collected from 0–15 cm and 15–30 cm depths before any field operations each year to determine the initial soil nutrient levels. The soil samples were air-dried, sieved through a 2 mm sieve, and then shipped to the Waters Agricultural Laboratories, Inc. in Camilla, GA, USA for analyses. Nitrate-N was measured with the automated flow injection analysis system (FIAlyzer-1000, FIAlab Instruments, Inc., Seattle, WA, USA) based on extraction in a 2 M KCl solution. Extractable P, K, Ca, Mg, S, Fe, Mn, Zn, B, and Cu were measured with an inductively coupled plasma optical emission spectrophotometer (ICP-OES; iCAP™ 6000 Series, Thermo Fisher Scientific, Cambridge, UK) after extraction in Mehlich I solution.

Plant tissue samples were collected within a uniform 1-m long strip of every plot by the six- to seven-leaf collar stage (V6 to V7 growth stage) and sent directly to Waters Agricultural Laboratories, Inc. in Camilla, GA for plant nutrient analyses. Plant samples at the V6-V7 growth stage were used because side-dress and other in-season nutrient applications are made around that stage or shortly after. Growers under non-irrigated conditions, or those who cannot inject fertilizers through their irrigation unit, cannot practically make nutrient applications after the V9-V10 stage. Thus, growers would need to account for the time it will take to have the laboratory results and the time to plan and apply nutrients, as may be needed.

For the nutrient analyses, plant samples were oven-dried to constant weight, pulverized, and sifted to <1 mm particle size. Total N was measured by dry combustion with LECO FP-528 Nitrogen Analyzer (LECO Corporation, St. Joseph, MI, USA). Total P, K, Ca, Mg, S, Fe, Mn, Zn, B, Cu, and Mo were measured with ICP-OES (iCAP™ 6000 Series, Thermo Fisher Scientific, Cambridge, UK) after digestion with DigiBLOC 3000 digestion system (SCP Science, Montreal, QC, Canada) with HNO_3_/H_2_O_2_ mixture.

Plant biomass was also determined for every plot by sampling aboveground plant samples within a uniform 1-m long strip of every plot at the same time that the plant tissue samples for nutrient analyses were collected. Weights of the aboveground plant samples were recorded after oven-drying at 78 °C to constant weight, and the values were used to compute plant biomass. In addition, relative biomass was calculated by dividing the biomass of every plot by the maximum biomass value obtained every year.

### 4.4. Statistical Analyses

The measured plant biomass and nutrient concentration data were analyzed with the linear mixed model using the ‘lme4’ package in R [45]. As the number of treatments and nutrient rates varied over the three years, separate analyses were performed for each year. Assumptions of normality of residuals and homoscedasticity were assessed, and data transformation was performed with the Box-Cox transformation or square root transformation methods as appropriate. Means generated from the analysis were separated using the least square means and the adjusted Tukey multiple comparison procedure with the ‘emmeans’ package in R [46]. Correlation matrices were computed for the biomass and nutrient concentrations using the ‘agricolae’ package in R [47]. The significance level for all analyses was assessed at *p* = 0.05.

## 5. Conclusions

The NSRs for corn at the early growth stage in the SCSB were not valid under the conditions tested in this study. Of the total samples, 47.6% satisfied all the lower thresholds of the NSRs. A 25.4% of samples had relative biomass <50%, with relative biomass even as low as 24.2% recorded in 2022. The poor growth of some samples could not be attributed to nutrient toxicity because no nutrient level was excessive. Moreover, 9.6% of the total samples had P or Cu that failed to meet the lower threshold but still had relative biomass ≥75%, suggesting either the lower thresholds of P and Cu are smaller than recommended or they were not the most limited. The findings of the study highlight the inability of the SRS to account for nutrient interactions and magnitude of the limitations of every nutrient at the V6 to V7 stage.

## Figures and Tables

**Table 1:** Nutrient application rates imposed in 2020, 2021, and 2022.

Year	Treatments	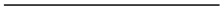 Nutrient Rates Applied (kg ha^−1^) 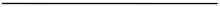											
		N	P_2_O_5_	K_2_O	Mg	Ca	S	B	Zn	Mn	Fe	Cu	Mo
2020	T2	121	110	112	11.2	11.2	11.2	0	0	0	0	0	0
	T3	121	110	112	0.0	0.0	0.0	0.56	2.24	3.36	2.24	0.56	0.28
	T4	121	110	112	11.2	11.2	11.2	0.56	2.24	3.36	2.24	0.56	0.28
	T6	242	197	224	11.2	11.2	11.2	0	0	0	0	0	0
	T7	242	197	224	0	0	0	0.56	2.24	3.36	2.24	0.56	0.28
	T8	242	197	224	11.2	11.2	11.2	0.56	2.24	3.36	2.24	0.56	0.28
	T10	363	330	280	16.8	16.8	16.8	1.12	3.36	6.73	4.48	1.12	0.56
	T12	0	0	0	0	0	0	0	0	0	0	0	0
2021	T1	135	101	101	0	0	0	0	0	0	0	0	0
	T2	135	101	101	5.60	11.2	5.60	0	0	0	0	0	0
	T3	135	101	101	0	0	0	0.56	1.12	3.36	2.24	0.28	0.28
	T4	135	101	101	5.60	11.2	5.60	0.56	1.12	3.36	2.24	0.28	0.28
	T5	247	168	191	0	0	0	0	0	0	0	0	0
	T6	247	168	191	5.60	11.2	5.60	0	0	0	0	0	0
	T7	247	168	191	0.00	0.0	0.00	0.56	1.12	3.36	2.24	0.28	0.28
	T8	247	168	191	5.60	11.2	5.60	0.56	1.12	3.36	2.24	0.28	0.28
	T9	146	168	168	5.60	5.6	5.60	0.56	1.12	3.36	2.24	0.28	0.28
	T10	269	202	202	11.2	11.2	16.8	1.12	2.24	6.73	4.48	0.56	0.56
	T11	224	213	269	0	0	11.2	0	0	0	0	0	0
	T12	0	0	0	0	0	0	0	0	0	0	0	0
2022	T1	179	157	146	0	0	0	0	0	0	0	0	0
	T2	179	157	146	5.60	5.60	11.2	0	0	0	0	0	0
	T3	179	157	146	0	0	0	0.56	3.36	5.60	2.24	0.56	0.28
	T4	179	157	146	5.60	5.60	11.2	0.56	3.36	5.60	2.24	0.56	0.28
	T5	291	224	235	0	0	0	0	0	0	0	0	0
	T6	291	224	235	5.60	5.60	11.2	0	0	0	0	0	0
	T7	291	224	235	0	0	0	0.56	3.36	5.60	2.24	0.56	0.28
	T8	291	224	235	5.60	5.60	11.2	0.56	3.36	5.60	2.24	0.56	0.28
	T9	54	49	0	0	0	0	0	0	0	0	0	0
	T10	359	314	291	11.2	11.2	16.8	1.12	5.04	8.41	3.36	1.12	0.56
	T11	202	224	258	0	0	11.2	0	3.36	0	0	0	0
	T12	0	0	0	0	0	0	0	0	0	0	0	0

**Table 2:** Average minimum, mean, and maximum temperatures, total growing degree days (GDD), solar radiation, and rainfall received from planting to tissue sampling in 2020, 2021, and 2022.

Year	Minimum Temperature	Mean Temperature	Maximum Temperature	GDD	Solar Radiation	Rainfall
	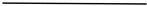 °C 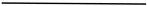				MJ m^−2^	mm
2020	13.6	19.8	26.0	304	668	144
2021	14.0	20.1	26.3	374	796	190
2022	12.0	18.6	25.1	291	716	83.8

GDD: Growing degree days calculated with a base temperature of 10 °C.

**Table 3:** Initial nutrient status of the experimental field soil at 0–15 cm and 15–30 cm depths in 2020, 2021, and 2022.

Year	Soil Depth	N	P	K	Mg	Ca	S	B	Zn	Mn	Fe	Cu
	cm	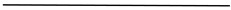 kg ha^−1^ 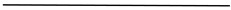										
2020	0–15	20.1	58.3	90.8	101	881	7.85	0.41	3.25	8.22	12.0	0.49
	15–30	13.3	38.1	74.7	63.9	582	15.3	0.30	3.18	7.47	13.8	0.49
2021	0–15	5.12	30.3	119	77.3	644	42.0	0.22	3.59	8.97	16.8	1.12
	15–30	3.82	24.7	103	71.7	600	15.1	0.22	3.64	6.16	15.7	2.63
2022	0–15	9.53	15.3	134	161	1076	12.3	0.37	2.09	7.10	28.8	0.26
	15–30	6.70	22.8	82.2	148	986	23.5	0.34	2.76	5.60	30.6	0.26

N was measured as Nitrate-N after extraction with 2 M KCl solution. Soil P, K, Ca, Mg, S, Fe, Mn, Zn, B, and Cu were measured after Mehlich I extraction.

**Table 4:** Summary statistics of the nutrients of samples with 95–100% relative biomass.

Statistics	N	P	K	Mg	Ca	S	B	Zn	Mn	Fe	Cu	Mo
	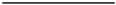 g kg^−1^ 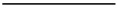						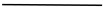 mg kg^−1^ 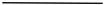					
Min	34.4	3.00	28.5	1.60	3.90	2.80	16.0	26.0	44.0	149	4.00	0.69
Mean	40.7	4.40	37.8	2.02	5.22	3.26	22.2	34.2	61.4	165	6.60	9.5
Max	48.4	6.20	50.6	2.60	6.60	4.30	40.0	45.0	99.0	182	10.0	29.0
SD	5.45	1.15	8.0	0.34	0.97	0.60	10.3	6.91	23.1	12.5	2.41	11.2

Min: Minimum; Max: Maximum; SD: Standard deviation.

**Table 5:** *p*-values after Pearson correlation of the relative biomass (RB) and nutrient concentrations.

	RB	N	P	K	Mg	Ca	S	B	Zn	Mn	Fe	Cu	Mo
RB	1												
N	<0.001	1											
P	0.004	<0.001	1										
K	0.001	0.021	<0.001	1									
Mg	0.694	<0.001	0.159	0.602	1								
Ca	0.464	<0.001	0.989	0.158	<0.001	1							
S	<0.001	<0.001	<0.001	<0.001	0.059	0.082	1						
B	0.045	<0.001	<0.001	0.005	0.79	0.319	<0.001	1					
Zn	0.003	<0.001	<0.001	<0.001	0.483	0.698	<0.001	<0.001	1				
Mn	0.02	<0.001	<0.001	<0.001	0.018	0.018	<0.001	0.01	<0.001	1			
Fe	0.505	0.052	0.879	0.089	<0.001	<0.001	0.755	0.098	0.3	<0.001	1		
Cu	0.103	<0.001	<0.001	<0.001	0.012	0.178	<0.001	<0.001	<0.001	<0.001	0.255	1	
Mo	0.006	<0.001	<0.001	0.002	0.100	0.236	0.006	<0.001	<0.001	0.201	0.901	<0.001	1

**Table 6:** Nutrient treatment effects on nutrient concentration of corn from 2020 to 2022.

Treatment	N	P	K	Mg	Ca	S	B	Zn	Mn	Fe	Cu	Mo
	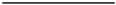 g kg^−1^ 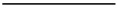						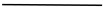 mg kg^−1^ 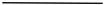					
	2020											
T2	46.0 a	5.47 a	49.17 a	2.97 b	7.57 a	4.33 b	17.7 a	39.7 a	108.0 a	207.0 a	9.33 a	3.5 c
T3	48.4 ab	6.50 ab	47.17 a	2.67 ab	6.53 a	3.50 a	34.3 ab	41.3 a	95.0 a	186.0 a	11.67 ab	64.1 a
T4	46.2 a	5.87 a	48.57 a	2.57 ab	6.37 a	4.00 ab	45.0 bc	43.7 a	280.3 a	261.3 a	14.00 ab	15.8 b
T6	49.7 ab	6.73 ab	49.20 a	2.83 ab	6.87 a	4.37 b	19.3 a	39.3 a	118.3 a	209.7 a	10.33 ab	1.0 c
T7	49.1 ab	7.60 b	48.63 a	2.30 a	6.17 a	3.63 a	42.7 bc	43.7 a	116.7 a	236.7 a	15.00 b	79.0 a
T8	49.7 ab	6.07 ab	50.03 a	2.67 ab	6.90 a	4.40 b	57.0 bc	56.7 b	139.0 a	206.0 a	21.67 c	26.9 ab
T10	52.7 b	5.37 a	43.83 a	2.30 a	5.80 a	4.07 ab	66.3 c	49.0 ab	123.7 a	185.0 a	15.00 b	13.3 b
T12 *	38.3	5.50	41.7	2.400	7.400	2.9	20.0	32.0	84.0	161	9.00	3.27
*p*-value	0.001	0.003	0.132	0.007	0.132	<0.001	<0.001	0.001	0.281	0.766	<0.001	<0.001
	2021											
T1	37.5 ab	3.52 a	33.50 a	1.95 a	6.20 a	2.80 ab	18.2 ab	30.0 a	56.0 a	181.8 a	4.25 ab	1.4 cd
T2	37.9 ab	3.48 a	33.35 a	1.95 a	5.42 a	2.80 ab	21.0 ab	30.5 a	47.8 a	165.8 a	4.00 ab	1.4 d
T3	37.2 ab	3.65 a	35.38 a	1.80 a	4.78 a	2.85 ab	23.2 ab	31.2 a	50.2 a	154.2 a	4.50 ab	5.6 ab
T4	35.6 ab	3.45 a	33.27 a	2.00 a	5.85 a	2.85 ab	24.2 ab	35.8 a	55.8 a	183.0 a	6.50 b	4.0 ab
T5	37.7 ab	3.50 a	33.77 a	1.90 a	5.70 a	2.75 ab	18.2 ab	29.0 a	46.0 a	166.8 a	4.50 ab	2.3 bcd
T6	37.4 ab	3.25 a	36.00 a	1.92 a	5.40 a	2.83 ab	18.8 ab	29.0 a	43.2 a	164.8 a	3.25 a	1.3 d
T7	37.5 ab	3.62 a	33.73 a	1.85 a	5.62 a	2.70 ab	29.2 a	30.2 a	53.8 a	162.8 a	4.25 ab	8.7 a
T8	37.2 ab	3.03 a	33.20 a	1.90 a	5.53 a	2.67 ab	23.2 ab	28.2 a	47.8 a	177.8 a	4.25 ab	2.8 abcd
T9	37.7 ab	3.28 a	33.80 a	2.02 a	6.42 a	3.02 b	27.0 a	33.8 a	56.2 a	183.2 a	5.25 ab	4.1 ab
T10	38.2 b	3.80 a	33.93 a	1.90 a	5.62 a	2.88 ab	25.5 a	31.2 a	53.0 a	176.2 a	4.50 ab	3.5 abc
T11	37.9 ab	3.65 a	32.73 a	1.95 a	5.95 a	2.72 ab	16.8 b	24.8 a	44.2 a	172.0 a	3.50 a	1.9 bcd
T12	32.1 a	3.25 a	31.48 a	2.10 a	6.58 a	2.53 a	23.0 ab	24.8 a	50.0 a	171.2 a	4.00 ab	2.1 bcd
*p*-value	0.046	0.658	0.112	0.318	0.068	0.052	0.001	0.017	0.321	0.4	0.012	<0.001
	2022											
T1	42.3 ab	3.03 bc	35.93 cd	2.87 a	6.80 a	2.37 a	16.0 ab	24.3 abc	68.3 a	266.7 c	2.33 a	5.5 a
T2	42.4 ab	2.98 b	37.53 d	2.62 a	6.68 a	2.55 ab	27.2 ab	23.5 abc	60.5 a	259.8 bc	4.75 abc	11.5 ab
T3	43.9 bc	2.98 b	39.08 d	2.67 a	6.78 a	2.45 a	21.2 ab	23.2 abc	62.8 a	225.8 abc	4.25 abc	15.7 abc
T4	51.0 d	3.38 bcd	25.68 b	3.22 a	7.65 a	3.00 bcd	36.0 abc	27.5 bc	60.0 a	171.2 a	8.00 c	42.3 c
T5	42.8 abc	3.25 bcd	37.05 d	2.90 a	6.68 a	2.43 a	10.5 ab	21.0 ab	60.2 a	282.8 c	3.25 ab	4.8 a
T6	41.9 ab	2.90 b	36.92 d	2.70 a	6.75 a	2.60 ab	18.2 ab	22.2 ab	60.2 a	290.2 c	5.50 abc	7.4 ab
T7	42.1 ab	3.05 bc	37.83 d	2.85 a	6.58 a	2.38 a	20.2 ab	22.0 ab	56.0 a	258.8 bc	5.00 abc	19.1 abc
T8	54.6 d	3.88 cd	27.42 bc	2.25 a	7.22 a	3.25 d	39.0 bc	35.2 c	81.2 a	178.5 ab	7.25 bc	32.9 bc
T9	43.8 bc	3.60 bcd	14.28 a	2.83 a	8.03 a	2.72 abc	3.5 a	25.0 abc	97.2 a	246.0 abc	5.75 abc	1.5 a
T10	51.0 d	4.00 d	26.92 b	2.65 a	6.43 a	3.20 cd	66.2 c	31.8 bc	71.0 a	162.8 a	7.75 bc	27.9 abc
T11	49.3 cd	4.00 d	30.95 bcd	2.53 a	8.07 a	3.10 cd	5.0 a	27.5 bc	70.2 a	206.0 abc	4.25 abc	1.8 a
T12	36.8 a	1.82 a	27.10 bc	2.55 a	6.68 a	2.28 a	4.2 a	15.5 a	21.5 b	206.5 abc	6.00 abc	3.8 a
*p*-value	<0.001	<0.001	<0.001	0.395	0.010	<0.001	<0.001	<0.001	<0.001	<0.001	0.001	<0.001

Within a study year, means not sharing any letter are significantly different using the least squares means and adjusted Tukey multiple comparisons (*p* < 0.05). * Only one replication, and so it was excluded from the statistical analyses.

**Table 7:** Fertilizers used as the primary sources of the different nutrient elements.

N	Urea; Ammonium nitrate; Urea ammonium nitrate solution (UAN 32)
P	Di-ammonium phosphate; triple super phosphate; Ammonium polyphosphate solution
K	Potassium chloride; Potassium nitrate
Mg	Magnesium oxy-sulfate; Magnesium nitrate solution
Ca	Calcium chloride; Calcium sulfate; Calcium nitrate solution
S	Potassium sulfate; Epsom; Ammonium thiosulfate solution
B	Fertilizer borate; Borosol^®^ 10 solution
Zn	Zinc oxysulfate; Zinc nitrate solution
Mn	Manganese sucrate; Manganese nitrate solution
Fe	Iron sucrate; chelated iron ethylenediaminetetraacetic acid; Iron nitrate solution
Cu	Copper sulfate; Copper nitrate solution
Mo	Sodium molybdate

## Data Availability

The data presented in this study are available on request from the corresponding author.

## Abbreviations

SRS, sufficiency range system; NSR, nutrient sufficiency range; CLA, critical level approach; DRIS, Diagnostic and Recommendation Integrated System; SCSB, Southern Cooperative Series Bulletin.
